# 2-Amino-5-bromo­pyridinium 3-amino­benzoate

**DOI:** 10.1107/S1600536810006288

**Published:** 2010-02-20

**Authors:** Madhukar Hemamalini, Hoong-Kun Fun

**Affiliations:** a X-ray Crystallography Unit, School of Physics, Universiti Sains Malaysia, 11800 USM, Penang, Malaysia

## Abstract

In the title salt, C_5_H_6_BrN_2_^+^·C_7_H_6_NO_2_^−^, the pyridine N atom of the 2-amino-5-bromo­pyridine mol­ecule is protonated. In the crystal, the protonated N atom and the 2-amino group are hydrogen-bonded to the carboxyl­ate O atoms *via* a pair of N—H⋯O hydrogen bonds, forming an *R*_2_^2^(8) ring motif. Two inversion-related 3-amino­benzoate anions are linked through N—H⋯O hydrogen-bonds, forming an *R*_2_^2^(14) ring motif. The crystal structure is further stabilized by π⋯π inter­actions involving the benzene and pyridinium rings with a centroid–centroid distance of 3.7743 (15) Å.

## Related literature

For background to the chemistry of substituted pyridines, see: Pozharski *et al.* (1997 ▶); Katritzky *et al.* (1996 ▶). Balasubramani & Fun (2009 ▶). For related structures, see: Goubitz *et al.* (2001 ▶); Vaday & Foxman (1999 ▶). For details of 3-amino­benzoic acid, see: Windholz (1976 ▶); Voogd *et al.* (1980 ▶). For details of hydrogen bonding, see: Jeffrey & Saenger (1991 ▶); Jeffrey (1997 ▶); Scheiner (1997 ▶). For hydrogen-bond motifs, see: Bernstein *et al.* (1995 ▶).
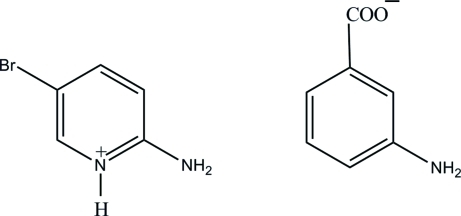


## Experimental

### 

#### Crystal data

C_5_H_6_BrN_2_^+^·C_7_H_6_NO_2_^−^
*M* *_r_* = 309.15
Monoclinic, 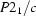

*a* = 10.1650 (7) Å
*b* = 11.0431 (7) Å
*c* = 11.9550 (9) Å
β = 113.710 (2)°
*V* = 1228.71 (15) Å^3^
*Z* = 4
Mo *K*α radiation
μ = 3.34 mm^−1^
*T* = 296 K
0.42 × 0.39 × 0.11 mm

#### Data collection

Bruker APEX DUO CCD area-detector diffractometer
Absorption correction: multi-scan (*SADABS*; Bruker, 2009 ▶) *T*_min_ = 0.332, *T*_max_ = 0.708
15251 measured reflections
3565 independent reflections
2650 reflections with *I* > 2σ(*I*)
*R*_int_ = 0.034

#### Refinement

*R*[*F* ^2^ > 2σ(*F* ^2^)] = 0.037
*wR*(*F* ^2^) = 0.137
*S* = 1.06
3565 reflections
163 parameters
H-atom parameters constrained
Δρ_max_ = 0.51 e Å^−3^
Δρ_min_ = −0.54 e Å^−3^

### 

Data collection: *APEX2* (Bruker, 2009 ▶); cell refinement: *SAINT* (Bruker, 2009 ▶); data reduction: *SAINT*; program(s) used to solve structure: *SHELXTL* (Sheldrick, 2008 ▶); program(s) used to refine structure: *SHELXTL*; molecular graphics: *SHELXTL*; software used to prepare material for publication: *SHELXTL* and *PLATON* (Spek, 2009 ▶).

## Supplementary Material

Crystal structure: contains datablocks global, I. DOI: 10.1107/S1600536810006288/tk2628sup1.cif

Structure factors: contains datablocks I. DOI: 10.1107/S1600536810006288/tk2628Isup2.hkl

Additional supplementary materials:  crystallographic information; 3D view; checkCIF report

## Figures and Tables

**Table 1:** Hydrogen-bond geometry (Å, °)

*D*—H⋯*A*	*D*—H	H⋯*A*	*D*⋯*A*	*D*—H⋯*A*
N1—H1⋯O1^i^	0.98	1.65	2.626 (3)	176
N2—H2*A*⋯O2^i^	0.86	1.99	2.826 (3)	165
N2—H2*B*⋯O1^ii^	0.86	2.06	2.909 (3)	170
N3—H3*B*⋯O2^iii^	0.86	2.26	3.028 (4)	148

Symmetry codes: (i) 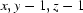; (ii) 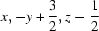; (iii) 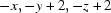.

**Fig. 1.**
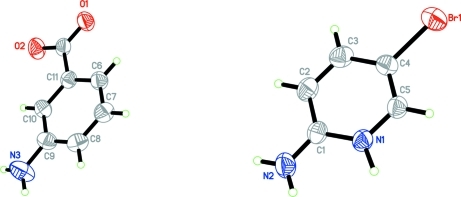
The asymmetric unit of (I) showing atom labelling scheme. Displacement ellipsoids are drawn at the 50% probability level.

**Fig. 2.**
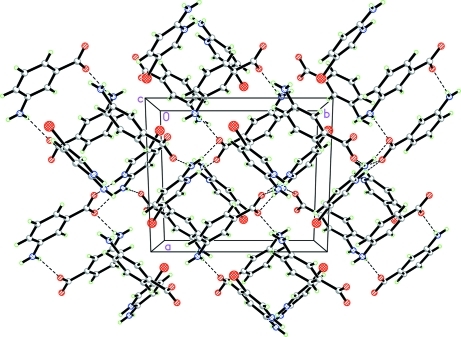
The crystal packing of (I), showing hydrogen-bonded (dashed lines) networks.

## supplementary crystallographic information

### Comment

Pyridine and its derivatives play an important role in heterocyclic chemistry (Pozharski *et al.*, 1997; Katritzky *et al.*, 1996). They are often involved in hydrogen-bond interactions (Jeffrey & Saenger, 1991; Jeffrey, 1997; Scheiner, 1997). 3-Aminobenzoic acid is used as an intermediate for dyes and pesticides (Windholz, 1976). The crystal structures of 3-aminobenzoic acid (Voogd *et al.*, 1980), 2-amino-5-bromopyridine (Goubitz *et al.*, 2001) and 2-amino-5-bromopyridinium propynoate (Vaday & Foxman, 1999) have been reported in the literature. In order to study some interesting hydrogen bonding interactions, the synthesis and structure of the title salt, (I), is presented here.

The asymmetric unit of (I) (Fig. 1) contains a 2-amino-5-bromopyridinium cation and a 3-aminobenzoate anion, indicating that proton transfer has occurred during the co-crystallisation experiment. In the 2-amino-5-bromopyridinium cation, a wider than normal angle (C5—N1—C1 = 122.5 (2)°) is subtented at the protonated N1 atom. The 2-amino-5-methylpyridinium cation is essentially planar, with a maximum deviation of 0.020 (2)Å for atom N1.

In the crystal packing (Fig. 2), the protonated N1 atom and 2-amino group (N2) are hydrogen-bonded to the carboxylate oxygen atoms (O1 and O2) via a pair of N—H···O hydrogen bonds forming a ring motif *R*_2_^2^(8) (Bernstein *et al.*, 1995). Two inversion-related 3-aminobenzoate anions are linked through N3—H3B···O2 hydrogen-bonding to form a *R*_2_^2^(14) ring motif (Table 1). This motif is also observed in the crystal structure of 2,3-diaminopyridinium 3-amino benzoate (Balasubramani & Fun, 2009). The crystal structure is further stabilized by a π···π stacking interaction between the pyridine rings (C1–C5/N1) and benzene ring (C6–C11) with a centroid- to-centroid distance of 3.7743 (15)Å [symmetry codes: 1-x, 1-y, 1-z].

### Experimental

A hot methanol solution (20 ml) of 2-amino-5-bromopyridine (87 mg, Aldrich) and 3-aminobenzoic acid (68 mg, Merck) were mixed and warmed over a heating magnetic stirrer for a few minutes. The resulting solution was allowed to cool slowly at room temperature and crystals of (I) appeared after a few days.

### Refinement

All hydrogen atoms were positioned geometrically [C–H = 0.93Å and N–H = 0.86–0.98Å] and were refined using a riding model, with *U*_iso_(H) = 1.2 *U*_eq_(C, N).

### Crystal data

**Table:**

C_5_H_6_BrN_2_^+^·C_7_H_6_NO_2_^−^	*F*(000) = 620
*M**_r_* = 309.15	*D*_x_ = 1.671 Mg m^−^^3^
Monoclinic, *P*2_1_/*c*	Mo *K*α radiation, λ = 0.71073 Å
Hall symbol: -P 2ybc	Cell parameters from 4215 reflections
*a* = 10.1650 (7) Å	θ = 2.6–26.8°
*b* = 11.0431 (7) Å	µ = 3.34 mm^−^^1^
*c* = 11.9550 (9) Å	*T* = 296 K
β = 113.710 (2)°	Blcok, brown
*V* = 1228.71 (15) Å^3^	0.42 × 0.39 × 0.11 mm
*Z* = 4	

### Data collection

**Table:**

Bruker APEX DUO CCD area-detector diffractometer	3565 independent reflections
Radiation source: fine-focus sealed tube	2650 reflections with *I* > 2σ(*I*)
graphite	*R*_int_ = 0.034
φ and ω scans	θ_max_ = 30.0°, θ_min_ = 2.2°
Absorption correction: multi-scan (*SADABS*; Bruker, 2009)	*h* = −14→14
*T*_min_ = 0.332, *T*_max_ = 0.708	*k* = −15→14
15251 measured reflections	*l* = −16→16

### Refinement

**Table:**

Refinement on *F*^2^	Primary atom site location: structure-invariant direct methods
Least-squares matrix: full	Secondary atom site location: difference Fourier map
*R*[*F*^2^ > 2σ(*F*^2^)] = 0.037	Hydrogen site location: inferred from neighbouring sites
*wR*(*F*^2^) = 0.137	H-atom parameters constrained
*S* = 1.06	*w* = 1/[σ^2^(*F*_o_^2^) + (0.0846*P*)^2^ + 0.125*P*] where *P* = (*F*_o_^2^ + 2*F*_c_^2^)/3
3565 reflections	(Δ/σ)_max_ = 0.001
163 parameters	Δρ_max_ = 0.51 e Å^−^^3^
0 restraints	Δρ_min_ = −0.54 e Å^−^^3^

### Special details

**Table:**

Geometry. All s.u.'s (except the s.u. in the dihedral angle between two l.s. planes) are estimated using the full covariance matrix. The cell s.u.'s are taken into account individually in the estimation of s.u.'s in distances, angles and torsion angles; correlations between s.u.'s in cell parameters are only used when they are defined by crystal symmetry. An approximate (isotropic) treatment of cell s.u.'s is used for estimating s.u.'s involving l.s. planes.
Refinement. Refinement of F^2^ against ALL reflections. The weighted R-factor wR and goodness of fit S are based on F^2^, conventional R-factors R are based on F, with F set to zero for negative F^2^. The threshold expression of F^2^ > 2σ(F^2^) is used only for calculating R-factors(gt) etc. and is not relevant to the choice of reflections for refinement. R-factors based on F^2^ are statistically about twice as large as those based on F, and R- factors based on ALL data will be even larger.

### Fractional atomic coordinates and isotropic or equivalent isotropic displacement parameters (Å^2^)

**Table:**

	*x*	*y*	*z*	*U*_iso_*/*U*_eq_	
Br1	0.82844 (3)	0.52926 (3)	0.04268 (3)	0.05749 (15)	
N1	0.5198 (2)	0.30649 (19)	0.06832 (17)	0.0383 (4)	
N2	0.4208 (3)	0.2756 (2)	0.2082 (2)	0.0499 (5)	
H2A	0.3715	0.2168	0.1638	0.060*	
H2B	0.4125	0.2940	0.2749	0.060*	
C1	0.5087 (2)	0.3373 (2)	0.1739 (2)	0.0378 (5)	
C2	0.5959 (3)	0.4328 (3)	0.2429 (2)	0.0442 (5)	
H2	0.5897	0.4569	0.3151	0.053*	
C3	0.6892 (3)	0.4899 (2)	0.2043 (3)	0.0448 (5)	
H3	0.7469	0.5525	0.2502	0.054*	
C4	0.6971 (2)	0.4537 (2)	0.0954 (2)	0.0387 (5)	
C5	0.6125 (2)	0.3619 (2)	0.0294 (2)	0.0387 (5)	
H5	0.6183	0.3370	−0.0428	0.046*	
O1	0.3733 (2)	1.13481 (18)	0.91776 (17)	0.0528 (5)	
O2	0.2423 (2)	1.11501 (19)	1.02651 (17)	0.0549 (5)	
N3	−0.0679 (3)	0.7483 (2)	0.8606 (3)	0.0677 (7)	
H3A	−0.1118	0.6865	0.8182	0.081*	
H3B	−0.0901	0.7746	0.9185	0.081*	
C6	0.2439 (3)	0.9284 (2)	0.7774 (2)	0.0426 (5)	
H6	0.3115	0.9693	0.7576	0.051*	
C7	0.1775 (3)	0.8250 (3)	0.7134 (2)	0.0477 (6)	
H7	0.2012	0.7963	0.6508	0.057*	
C8	0.0755 (3)	0.7644 (2)	0.7426 (2)	0.0461 (6)	
H8	0.0315	0.6954	0.6992	0.055*	
C9	0.0388 (3)	0.8058 (2)	0.8355 (2)	0.0445 (5)	
C10	0.1058 (3)	0.9094 (2)	0.9004 (2)	0.0412 (5)	
H10	0.0820	0.9380	0.9630	0.049*	
C11	0.2085 (3)	0.9701 (2)	0.8712 (2)	0.0375 (5)	
C12	0.2807 (3)	1.0815 (2)	0.9441 (2)	0.0399 (5)	
H1	0.4617	0.2439	0.0129	0.048*	

### Atomic displacement parameters (Å^2^)

**Table:**

	*U*^11^	*U*^22^	*U*^33^	*U*^12^	*U*^13^	*U*^23^
Br1	0.0532 (2)	0.0605 (2)	0.0637 (2)	−0.01495 (12)	0.02867 (15)	−0.00314 (12)
N1	0.0453 (10)	0.0357 (10)	0.0354 (9)	−0.0041 (8)	0.0179 (8)	−0.0056 (8)
N2	0.0630 (14)	0.0513 (13)	0.0451 (11)	−0.0075 (10)	0.0320 (10)	−0.0064 (10)
C1	0.0432 (11)	0.0364 (12)	0.0346 (10)	0.0035 (9)	0.0165 (8)	−0.0018 (9)
C2	0.0519 (13)	0.0432 (13)	0.0391 (12)	0.0008 (11)	0.0200 (10)	−0.0102 (10)
C3	0.0454 (13)	0.0388 (12)	0.0480 (13)	−0.0018 (10)	0.0167 (10)	−0.0113 (10)
C4	0.0357 (11)	0.0374 (12)	0.0438 (12)	−0.0006 (8)	0.0168 (9)	−0.0014 (9)
C5	0.0427 (11)	0.0401 (13)	0.0356 (11)	−0.0004 (9)	0.0183 (9)	−0.0031 (9)
O1	0.0679 (12)	0.0533 (12)	0.0469 (10)	−0.0214 (9)	0.0332 (9)	−0.0119 (8)
O2	0.0784 (13)	0.0500 (11)	0.0479 (10)	−0.0120 (9)	0.0376 (10)	−0.0104 (8)
N3	0.0686 (16)	0.0571 (16)	0.094 (2)	−0.0195 (13)	0.0496 (15)	−0.0166 (15)
C6	0.0455 (12)	0.0433 (13)	0.0412 (12)	−0.0013 (10)	0.0196 (9)	−0.0006 (10)
C7	0.0490 (13)	0.0480 (15)	0.0467 (13)	0.0038 (11)	0.0198 (10)	−0.0063 (11)
C8	0.0443 (13)	0.0380 (13)	0.0508 (14)	0.0026 (10)	0.0137 (10)	−0.0052 (11)
C9	0.0384 (11)	0.0407 (13)	0.0546 (14)	0.0005 (9)	0.0191 (10)	0.0024 (11)
C10	0.0430 (12)	0.0389 (13)	0.0457 (12)	0.0005 (9)	0.0221 (10)	0.0008 (10)
C11	0.0422 (11)	0.0334 (12)	0.0353 (11)	0.0024 (8)	0.0139 (9)	0.0034 (9)
C12	0.0493 (13)	0.0365 (12)	0.0336 (10)	−0.0017 (9)	0.0163 (9)	0.0021 (9)

### Geometric parameters (Å, °)

**Table:**

Br1—C4	1.885 (2)	O2—C12	1.252 (3)
N1—C5	1.353 (3)	N3—C9	1.390 (3)
N1—C1	1.355 (3)	N3—H3A	0.8600
N1—H1	0.9745	N3—H3B	0.8600
N2—C1	1.313 (3)	C6—C11	1.387 (3)
N2—H2A	0.8600	C6—C7	1.389 (4)
N2—H2B	0.8600	C6—H6	0.9300
C1—C2	1.410 (4)	C7—C8	1.391 (4)
C2—C3	1.365 (4)	C7—H7	0.9300
C2—H2	0.9300	C8—C9	1.384 (4)
C3—C4	1.395 (4)	C8—H8	0.9300
C3—H3	0.9300	C9—C10	1.396 (4)
C4—C5	1.358 (3)	C10—C11	1.398 (3)
C5—H5	0.9300	C10—H10	0.9300
O1—C12	1.254 (3)	C11—C12	1.515 (3)
			
C5—N1—C1	122.5 (2)	H3A—N3—H3B	120.0
C5—N1—H1	113.7	C11—C6—C7	119.5 (2)
C1—N1—H1	123.8	C11—C6—H6	120.3
C1—N2—H2A	120.0	C7—C6—H6	120.3
C1—N2—H2B	120.0	C6—C7—C8	120.2 (2)
H2A—N2—H2B	120.0	C6—C7—H7	119.9
N2—C1—N1	118.8 (2)	C8—C7—H7	119.9
N2—C1—C2	123.5 (2)	C9—C8—C7	120.8 (2)
N1—C1—C2	117.7 (2)	C9—C8—H8	119.6
C3—C2—C1	120.4 (2)	C7—C8—H8	119.6
C3—C2—H2	119.8	C8—C9—N3	120.6 (2)
C1—C2—H2	119.8	C8—C9—C10	119.2 (2)
C2—C3—C4	119.5 (2)	N3—C9—C10	120.2 (2)
C2—C3—H3	120.3	C9—C10—C11	120.1 (2)
C4—C3—H3	120.3	C9—C10—H10	120.0
C5—C4—C3	119.7 (2)	C11—C10—H10	120.0
C5—C4—Br1	120.33 (18)	C6—C11—C10	120.3 (2)
C3—C4—Br1	119.97 (19)	C6—C11—C12	120.8 (2)
N1—C5—C4	120.3 (2)	C10—C11—C12	118.9 (2)
N1—C5—H5	119.9	O2—C12—O1	124.2 (2)
C4—C5—H5	119.9	O2—C12—C11	117.4 (2)
C9—N3—H3A	120.0	O1—C12—C11	118.4 (2)
C9—N3—H3B	120.0		
			
C5—N1—C1—N2	−177.0 (2)	C7—C8—C9—N3	176.9 (3)
C5—N1—C1—C2	1.5 (3)	C7—C8—C9—C10	−0.2 (4)
N2—C1—C2—C3	177.4 (3)	C8—C9—C10—C11	0.0 (4)
N1—C1—C2—C3	−1.0 (4)	N3—C9—C10—C11	−177.1 (3)
C1—C2—C3—C4	0.4 (4)	C7—C6—C11—C10	−0.7 (4)
C2—C3—C4—C5	−0.2 (4)	C7—C6—C11—C12	179.1 (2)
C2—C3—C4—Br1	−178.6 (2)	C9—C10—C11—C6	0.4 (4)
C1—N1—C5—C4	−1.3 (4)	C9—C10—C11—C12	−179.3 (2)
C3—C4—C5—N1	0.7 (4)	C6—C11—C12—O2	179.1 (2)
Br1—C4—C5—N1	178.99 (18)	C10—C11—C12—O2	−1.2 (3)
C11—C6—C7—C8	0.5 (4)	C6—C11—C12—O1	−0.2 (4)
C6—C7—C8—C9	−0.1 (4)	C10—C11—C12—O1	179.5 (2)

### Hydrogen-bond geometry (Å, °)

**Table:**

*D*—H···*A*	*D*—H	H···*A*	*D*···*A*	*D*—H···*A*
N1—H1···O1^i^	0.98	1.65	2.626 (3)	176
N2—H2A···O2^i^	0.86	1.99	2.826 (3)	165
N2—H2B···O1^ii^	0.86	2.06	2.909 (3)	170
N3—H3B···O2^iii^	0.86	2.26	3.028 (4)	148

Symmetry codes: (i) *x*, *y*−1, *z*−1; (ii) *x*, −*y*+3/2, *z*−1/2; (iii) −*x*, −*y*+2, −*z*+2.
